# Tauroursodeoxycholic Acid Protects Retinal and Visual Function in a Mouse Model of Type 1 Diabetes

**DOI:** 10.3390/pharmaceutics13081154

**Published:** 2021-07-27

**Authors:** Jieming Fu, Moe H. Aung, Megan C. Prunty, Adam M. Hanif, Lauren M. Hutson, Jeffrey H. Boatright, Machelle T. Pardue

**Affiliations:** 1 Center for Visual and Neurocognitive Rehabilitation, Atlanta VA Medical Center, Decatur, GA 30033, USA; jf4ad@virginia.edu (J.F.); kyawhein@gmail.com (M.H.A.); megan.prunty1@gmail.com (M.C.P.); hanif@ohsu.edu (A.M.H.); laurhut@live.unc.edu (L.M.H.); 2 Neuroscience Graduate Program, University of Virginia, Charlottesville, VA 22904, USA; 3 Neuroscience, Emory University, Atlanta, GA 30322, USA; 4 Department of Ophthalmology, Dell Medical School, The University of Texas at Austin, Austin, TX 78712, USA; 5 Case Western Reserve University School of Medicine, Urology Institute of University Hospitals, Case Western Reserve University, Cleveland, OH 44106, USA; 6 Casey Eye Institute, Oregon Health and Science University, Portland, OR 97239, USA; 7 Department of Psychology and Neuroscience, The University of North Carolina at Chapel Hill, Chapel Hill, NC 27514, USA; 8 Ophthalmology, Emory University, Atlanta, GA 30322, USA; 9 Wallace H. Coulter Department of Biomedical Engineering, Georgia Institute of Technology and Emory School of Medicine, Atlanta, GA 30332, USA

**Keywords:** diabetic retinopathy, tauroursodeoxycholic acid, retina, mouse model, electroretinogram, optomotor response

## Abstract

Purpose: Previous studies demonstrated that systemic treatment with tauroursodeoxycholic acid (TUDCA) is protective in in vivo mouse models of retinal degeneration and in culture models of hyperglycemia. This study tested the hypothesis that TUDCA will preserve visual and retinal function in a mouse model of early diabetic retinopathy (DR). Methods: Adult C57BL/6J mice were treated with streptozotocin (STZ) and made diabetic at 8–10 weeks of age. Control and diabetic mice were treated with vehicle or TUDCA starting 1 or 3 weeks after induction of diabetes, and were assessed bimonthly for visual function via an optomotor response and monthly for retinal function via scotopic electroretinograms. Results: Diabetic mice showed significantly reduced spatial frequency and contrast sensitivity thresholds compared to control mice, while diabetic mice treated early with TUDCA showed preservation at all timepoints. A-wave, b-wave, and oscillatory potential 2 (OP2) amplitudes decreased in diabetic mice. Diabetic mice also exhibited delays in a-wave and OP2-implicit times. Early TUDCA treatment ameliorated a-wave, b-wave, and OP2 deficits. Late TUDCA treatment showed reduced preservation effects compared to early treatment. Conclusions: Early TUDCA treatment preserved visual function in an STZ-mouse model of Type I diabetes. These data add to a growing body of preclinical research that may support testing whether TUDCA may be an effective early clinical intervention against declining visual function caused by diabetic retinopathy.

## 1. Introduction

Diabetic retinopathy (DR) is a common late-stage complication of diabetes mellitus (DM) and is the leading cause of blindness among working-age adults in the United States [1]. According to the National Eye Institute, the number of adults with DR is projected to double to 14 million by 2050 [2]. Diabetic retinopathy results in a loss of vision and is clinically diagnosed via an observation of vascular abnormalities in the retina. Typical treatments include ocular laser photocoagulation and intraocular injections of drugs that block the activity of the vascular endothelial growth factor (VEGF), both of which target excess vascular endothelial growth in the retina. However, neuronal damage precedes vascular damage in DR [3,4,5,6,7,8], and deficits in visual function are often irreversible by the time retinal vasculature is visibly compromised. Thus, it is crucial that future approaches to DR include early detection and neuroprotective treatment to address early stage functional changes in DR.

Tauroursodeoxycholic acid (TUDCA) is a hydrophilic bile acid that is neuroprotective across a wide range of disease models, including heart disease and neurodegenerative disorders [9,10,11,12,13,14,15]. Systemic delivery of TUDCA is protective against photoreceptor cell death and retinal function loss in in vivo rodent models of retinal degeneration [16,17,18,19,20,21,22,23,24,25,26,27], while oral dosing with UDCA, the parent conjugate of TUDCA, improves patient visual outcomes following retinal detachment surgery [28]. TUDCA acts through multiple neuroprotective mechanisms, including reducing oxidative and ER stress in models of myocardial dysfunction [9,12] and models of Parkinson’s disease [11]. TUDCA also suppresses apoptosis through the stabilization of the mitochondrial membrane, preventing the translocation of apoptosis-inducing factors to the nucleus [29]. Finally, and specific to DR, it has been proposed that the protective effects of intermittent fasting in an in vivo mouse model of DR are in part mediated by the elevation of circulating levels of TUDCA activating the Takeda G-protein-coupled receptor 5 (TGR5) in the retina [30].

The pleiotropic variety of mechanisms affected by TUDCA make it a good candidate for neuroprotection in the early stages of a disease such as diabetic retinopathy, where functional outcomes may be very sensitive to change, but identifying specific cellular pathways to treat has proven difficult. Indeed, TUDCA treatment of retinal cell and tissue culture models of hyperglycemia decreases apoptosis, mitochondrial apoptosis factor translocation, and oxidative stress as well as increases retinal cell survival and the neurite number [29,31] (Reviewed in [27]). However, little is known of the effect of systemic TUDCA treatment in in vivo DR models, especially functional outcomes. Thus, in this study, we sought to test our hypothesis that TUDCA will be an effective neuroprotective treatment against early retinal and visual function deficits caused by diabetes as both an early supplementary therapy for diabetes mellitus and a late interventional treatment.

## 2. Methods

### 2.1. Animals

All animal procedures were approved by the Institution for Animal Care and Use Committee at the Atlanta VA Medical Center (V010-16, approved 8 June 2016) and conform to the Association for Research in Vision and Ophthalmology (ARVO) Statement for the Use of Animals in Ophthalmic and Vision Research and the National Institutes of Health guide for the care and use of laboratory animals (NIH Publications No. 8023, revised 1978).

Male and female C57BL/6J mice (*n* = 121) between 8 and 10 weeks of age (The Jackson Laboratory, Bar Harbor, ME, USA) were housed on a 12 h light/dark cycle and given food and water ad libitum. A subset of mice (*n* = 70) were made diabetic with intraperitoneal injection of streptozotocin (STZ: 50 mg/kg; Sigma-Aldrich, St. Louis, MO, USA) dissolved in 8:1 citrate buffer and 50% glucose solution and administered in sequential small doses over the course of 5 days, as previously described [32]. DM was defined as two successive blood sugar levels at or above 250 mg/dL. Animals were monitored for weight and blood glucose twice per week, and those that lost more than 10% body weight in less than one week received subcutaneous insulin injection (Novo Nordisk Inc., Plainsboro, NJ, USA,). The group of mice not made diabetic is referred to as “Ctrl” and the group that was made diabetic is referred to as “DM.”

Mice were further divided into two subgroups to test the effects of different treatment timing. Mice in the preventative treatment group (early treatment) received TUDCA or vehicle 1 week after the onset of hyperglycemia, while mice in the interventional treatment group (late treatment) received TUDCA or vehicle 3 weeks after hyperglycemia was confirmed. TUDCA-treated mice were given TUDCA (500 mg/kg; VWR, Radnor, PA, USA) dissolved in 0.15M NaHCO_3_ twice per week, and vehicle-treated mice were given bicarbonate buffer solution only. In total, this experiment tracked four different treatment groups (Ctrl+Veh, Ctrl+TUDCA, DM+Veh, DM+TUDCA) across two different treatment timings (early, late) for a total of eight different experimental groups.

Starting from 4 weeks after initial administration of STZ, mice were assessed every two weeks for visual acuity and contrast sensitivity using optomotor response (OMR), and every four weeks for retinal function using flash electroretinogram (ERG) over the 10-week study period.

### 2.2. Assessing Visual Function via Optomotor Response

Visual function was measured using a virtual optomotor tracking system (OptoMotry, CerebralMechanics, Lethbridge, AB, Canada) as previously described [33,34,35]. Animals were placed on an elevated platform within a testing chamber composed of four computer monitors and shown a virtual spatial frequency grating that rotated laterally. Animals were then observed for OMR via a camera located in the ceiling of the testing chamber. A staircase paradigm was used to alter the spatial frequency or contrast sensitivity until the minimum threshold stimulus required to elicit the optomotor response could be determined. Gratings were rotated clockwise or counterclockwise to independently assess visual function in the left and right eyes [33]. Spatial frequency was measured at 100% contrast, and contrast sensitivity was measured at the spatial frequency of 0.102 cyc/deg; these parameters have been demonstrated in the literature to elicit responses of the maximum sensitivity [6]. A positive OMR was defined as a lateral head movement in the same direction as grating rotation.

During analysis, the threshold values of left and right eyes were averaged to produce a representative value for each animal. Contrast sensitivity was calculated as a reciprocal of the Michelson contrast based on screen luminance, as previously described [6,33,34,35].

### 2.3. Assessing Retinal Function

Retinal function was assessed using flash electroretinography (ERG) as previously described [32,36,37]. Mice were dark-adapted overnight. Prior to recording, mice were anesthetized with intraperitoneal ketamine (65 mg/kg)/xylazine (9.9 mg/kg) (Vedco, Inc, Saint Joseph, MI, USA). Eyes received drops of tetracaine (0.5%; Alcon, Fort Worth, TX, USA) for corneal anesthesia and tropicamide (1%, Sandoz Inc., Princeton, NY, USA) for pupil dilation.

Retinal responses were recorded via gold loop electrodes placed in contact with the cornea via a layer of topical methylcellulose (1%, Allergan Inc., Irvine, CA, USA), referenced against 1-cm platinum electrodes inserted subcutaneously in the cheek, and grounded to an additional platinum electrode inserted in the tail. The ERG stimulus consisted of 5 scotopic full-field flashes of increasing intensity (−3.0 to 2.1 log cd s/m^2^), with intervals between scotopic flashes adjusted to the brightness of each step, ranging from 2 s to 70 s.

After testing, animals were given intraperitoneal injections of atipamezole (Antisedan 0.4 mg/kg; Zoetis, Parsippany, NJ, USA) to reverse anesthetic effects of xylazine, and topical ointment (Petrolatum, Dechra Veterinary Products, Overland Park, KS, USA) to prevent corneas from drying out during recovery.

ERG traces were saved and analyzed using commercial ERG software (UTAS-3000; LKC Technologies, Gaithersburg, MD, USA). Traces were analyzed for a-waves, b-waves, and oscillatory potentials (OPs), which are, respectively the responses from photoreceptors [38], depolarizing bipolar cells [39], and amacrine cells [40]. A-wave amplitude was measured from baseline to the trough of the first negative wave, and b-wave amplitude was measured from the a-wave trough to the apex of the first positive wave. In the absence of an a-wave, b-waves were measured from baseline to the apex of the first positive wave. All implicit times were measured from flash to peak. Raw traces were filtered (75–500 Hz) for oscillatory potentials (OPs), which were then measured from preceding trough to peak for amplitude and implicit time. Amplitude and implicit time from left and right eye traces were averaged for each individual animal to produce a combined value for statistical analysis [36].

### 2.4. Statistical Analysis

Data processing and cleaning was performed using R (R Foundation for Statistical Computing, Vienna, Austria) and two-way ANOVA, and two-way repeated measures ANOVA were performed using SigmaStat (Systat Software, Inc., Chicago, IL, USA). Significance was set at α = 0.05 for all tests. All values are presented as mean plus or minus SEM. Where applicable, Holm–Sidak post hoc comparisons are indicated on figures with asterisks.

## 3. Results

### 3.1. Effects of DM and TUDCA Treatment on Body Weight and Blood Glucose

**Figure 1:**
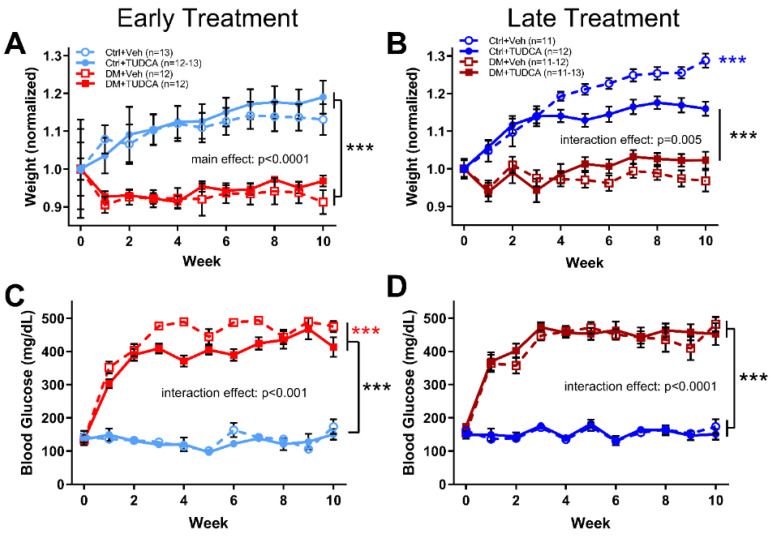
Effects of DM and TUDCA treatment on body weight and blood glucose. (**A**) Early treatment body weight, normalized to mean baseline (0 week timepoint) weight by group. As animals aged, the Ctrl groups weighed significantly more than the DM groups (two-way ANOVA, F_3,506_ = 1.831, main effect *p* < 0.0001). (**B**) Late treatment body weight, normalized to baseline by group at 0-week timepoint. Again, the DM groups weighed significantly less than the Ctrl groups. However, the Ctrl + TUDCA group also weighed less than the Ctrl+Veh group (*** *p* < 0.0001) (two-way ANOVA, F_30,484_ = 4.330, interaction effect *p* < 0.0001). (**C**) In the early treatment experiment, blood glucose levels were significantly higher in both DM groups compared to Ctrl groups (two-way ANOVA, F_30,506_ = 15.19, interaction effect *p* < 0.0001). In addition, the DM+TUDCA group had significantly lower blood glucose than the DM+Veh group (*** *p* < 0.0001). (**D**) In the late treatment experiments, blood glucose in the DM groups was significantly higher than the Ctrl groups (two-way ANOVA, F_30,484_ = 7.604, interaction effect *p* < 0.0001). Holm–Sidak post hoc comparisons indicated by asterisks.

All groups had statistically indistinguishable mean weights at baseline, but following STZ treatment, all early DM animals (either given vehicle or TUDCA) weighed less than Ctrl animals (Figure 1A, two-way ANOVA, F_3,506_ = 56.31, main effect *p* < 0.0001). Late TUDCA treatment resulted in lower average body weight within Ctrl animals but did not significantly alter weight amongst the DM mice (Figure 1B, two-way ANOVA, F_30,484_ = 4.330, interaction effect *p* < 0.0001). In both early and late treatment groups, all DM animals were significantly more hyperglycemic (blood glucose >250 mg/dL) than their Ctrl counterparts throughout the course of the study (Figure 1C, early treatment two-way ANOVA, F_30,506_ = 15.19, interaction effect *p* < 0.0001; Figure 1D, late treatment two-way ANOVA, F_30,484_ = 7.604, interaction effect *p* < 0.0001). Interestingly, early TUDCA treatment lowered blood glucose levels in DM animals when comparing between DM=Veh and DM+TUDCA groups (Figure 1C, *p* < 0.001).

### 3.2. TUDCA Protects Visual Function in Diabetic Animals

**Figure 2:**
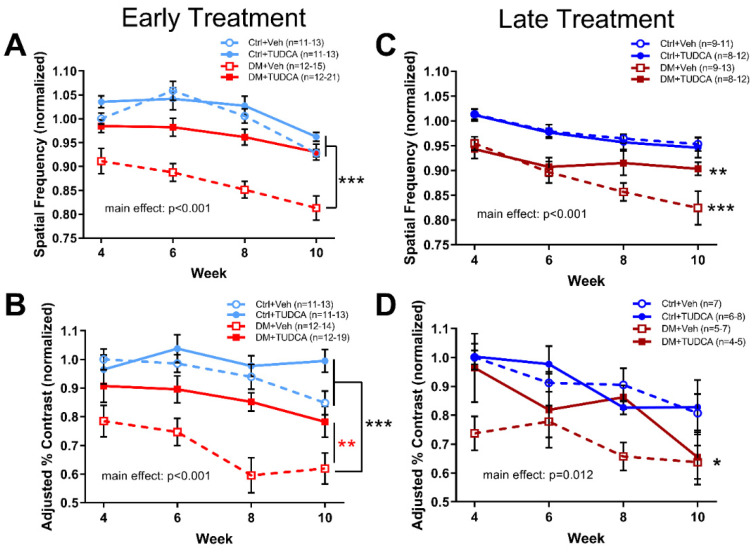
Early TUDCA treatment preserves visual function in diabetic mice. All groups were assessed for spatial frequency and contrast sensitivity thresholds at 4, 6, 8 and 10 weeks following induction of hyperglycemia with values normalized to Ctrl+Veh at 4 weeks. (**A**) In the early treatment experiment, spatial frequency thresholds for Ctrl+Veh, Ctrl+TUDCA, and DM+TUDCA groups were similar, and were significantly higher than those of the DM+Veh group (two-way repeated measures ANOVA, F_3,212_ = 37.94, main effect of treatment *p* < 0.001). (**B**) In the late treatment experiment, spatial frequency thresholds for Ctrl+Veh and Ctrl+TUDCA groups were similar and significantly higher than those of DM+Veh (*** *p* < 0.001) and DM+TUDCA groups (** *p* = 0.008) (two-way repeated measures ANOVA, F_3,170_=17.58, main effect of treatment *p* < 0.001). (**C**) In the early treatment experiment, contrast sensitivity thresholds for Ctrl+Veh and Ctrl+TUDCA groups were similar and both were significantly higher than the DM+Veh group (two-way repeated measures ANOVA, F_3,207_ = 16.07, main effect of treatment, *p* < 0.001). The DM+TUDCA group also significantly differed from DM+Veh (** *p* = 0.004). (**D**) In the late treatment experiment, contrast sensitivity thresholds for the Ctrl+Veh group were significantly higher than those of the DM+Veh group (* *p* = 0.026; two-way repeated measures ANOVA, F_3,95_ = 4.42, main effect *p* = 0.012). Holm–Sidak post hoc comparisons indicated by asterisks.

Animals were assessed for visual function via OMR every two weeks, starting at 4 weeks after hyperglycemia. DM+Veh animals in either the early or late treatment paradigm had significantly decreased spatial frequency thresholds (Figure 2A, early treatment, two-way repeated measures ANOVA, F_3,212_ = 37.94, main effect of treatment *p* < 0.001; Figure 2B, late treatment, two-way repeated measures ANOVA, F_3,170_ = 17.58, main effect of treatment *p* < 0.001) and contrast sensitivity thresholds (Figure 2C, early treatment, two-way repeated measures ANOVA, F_3,207_ = 16.07, main effect of treatment *p* < 0.001; Figure 2D, late treatment, two-way repeated measures ANOVA, F_3,95_ = 4.42, main effect of treatment *p* = 0.012) compared to non-diabetic controls (Ctrl+Veh, Ctrl+TUDCA). Early treatment with TUDCA protected visual function in diabetic animals such that early treatment DM+TUDCA animals were statistically indistinguishable from non-diabetic controls in both the spatial frequency and contrast sensitivity thresholds (Figure 2A,C). Late treatment with TUDCA had a smaller protective effect such that DM+TUDCA animals were statistically different from controls for spatial frequency thresholds (Figure 2B), but not contrast sensitivity (Figure 2D).

### 3.3. TUDCA Protects Retinal Function in Diabetic Animals

**Figure 3:**
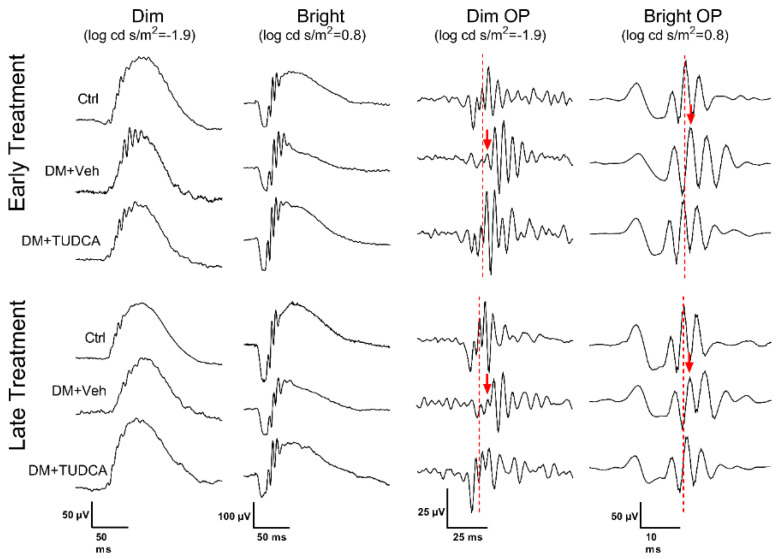
Representative waveforms from dim (log cd s/m^2^ = −1.9) and bright (log cd s/m^2^ = 0.8) stimuli, as well as filtered OPs, at week 8 post-STZ for both early and late treatment. Dashed lines indicate the peak of OP2 in the Ctrl waveform, whereas the red arrows indicate the delayed peak of OP2 in the DM+Veh animals.

**Figure 4:**
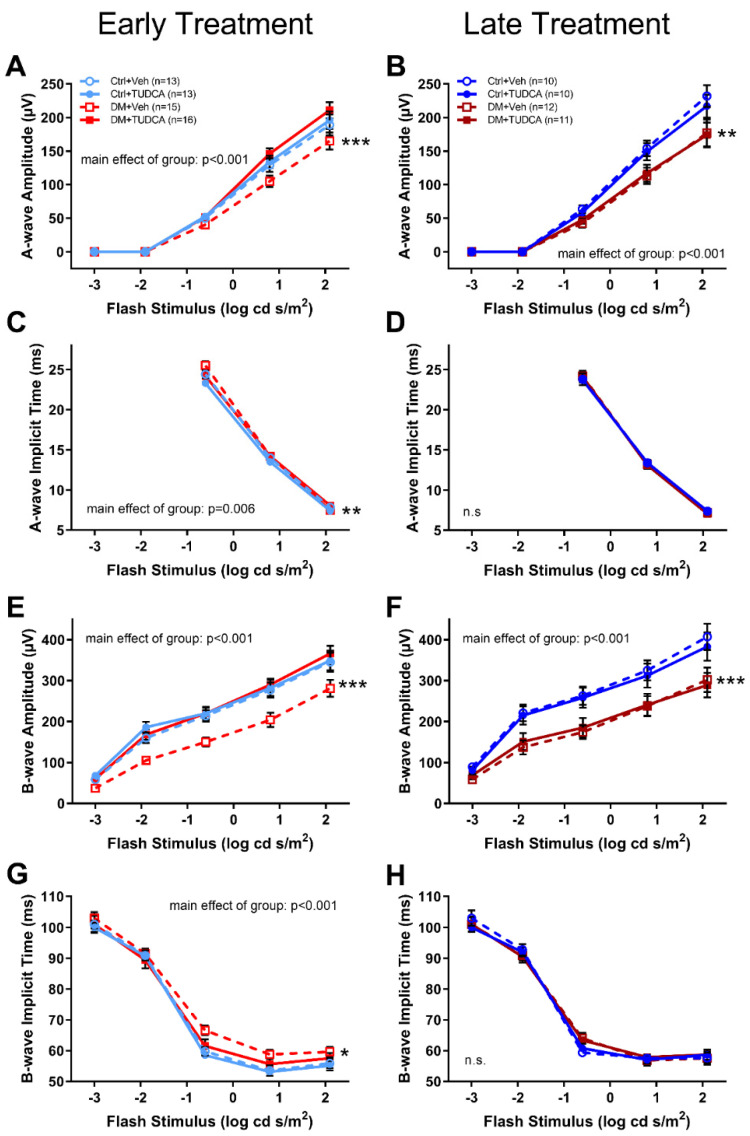
Early TUDCA treatment preserved ERG a- and b-waves in diabetic mice by 8 weeks post-STZ. (**A**) With early treatment, a-wave amplitudes in the DM+Veh group were significantly reduced compared to the DM+Veh, Ctrl+Veh, Ctrl+TUDCA groups (*** *p* < 0.001; two-way repeated measures ANOVA, F_12,298_ = 4.121, main effect *p* < 0.001). (**B**) With late treatment, a-wave amplitudes were significantly reduced in the DM groups compared to the Ctrl groups (** *p* < 0.01; two-way repeated measures ANOVA, F_12,243_ = 2.416, main effect of group *p* < 0.001). (**C**) With early treatment, a-wave implicit times were delayed in the DM+Veh group compared to all other groups (** *p* < 0.01; two-way repeated measures ANOVA, F_3,170_ = 3.34, main effect of group *p* = 0.006). (**D**) With late treatment, a-wave implicit times were not significantly different. (**E**) With early treatment, b-wave amplitudes in the DM versus Ctrl groups (*** *p* < 0.001; two-way repeated measures ANOVA, F_12,282_ = 2.421, main effect of group *p* < 0.001). (**F**) With late treatment, b-wave amplitudes were reduced in the DM+Veh group compared to the other groups (two-way repeated measures ANOVA, F_12,219_ = 2.416, main effect *p* < 0.001). (**G**) With early treatment, b-wave implicit times in the DM+Veh group were significantly delayed compared to the other groups (two-way repeated measures ANOVA, F_12,282_ = 2.46, main effect *p* < 0.001). (**H**) With late treatment, b-wave implicit times showed no significant differences. Holm–Sidak post hoc comparisons indicated by asterisks: * *p* < 0.05, ** *p* < 0.01, *** *p* < 0.001.

**Figure 5:**
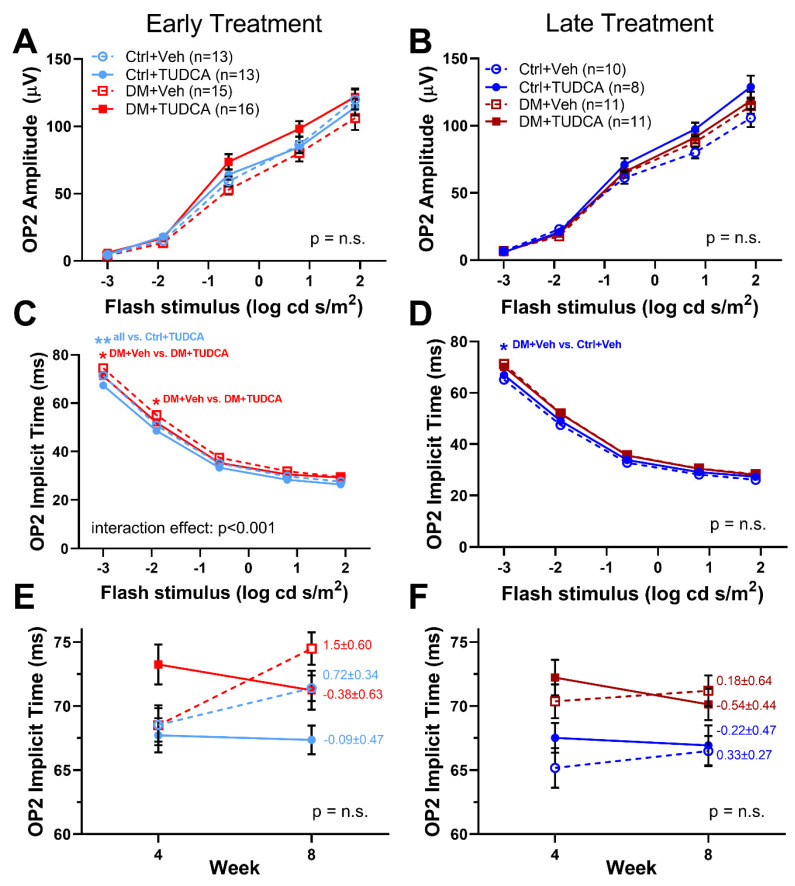
Early TUDCA treatment preserved ERG OPs in diabetic mice. (**A**) With early treatment, OP2 amplitudes at 8 weeks post-STZ were similar between groups. (**B**) With late treatment, OP2 amplitudes at 8 weeks post-STZ were similar between all treatment groups. (**C**) With early treatment, OP2 implicit times at 8 weeks were significantly delayed in the DM+Veh group at the two dimmest flash stimuli compared to DM+TUDCA (two-way repeated measures ANOVA F_12,212_ = 3.541, interaction effect *p* < 0.001). (**D**) With late treatment, OP2 implicit times were similar for all groups. (**E**) With early treatment, OP2 implicit times were plotted for 4 and 8 weeks post-STZ and the slope of the change calculated. While the DM+Veh group had a positive slope, the DM+TUDCA group was negative. (**F**) In the late treatment group, the slope of the OP2 implicit times were reduced, but still showed a similar trend, with the slope of the DM+Veh group having positive values and the DM+TUDCA group having negative values. Holm–Sidak post hoc comparisons indicated by asterisks: * *p* <0.05, ** *p* <0.01; n.s. = not significant.

ERGs were assessed on all animals at 4 and 8 weeks following induction of hyperglycemia. Representative ERG traces at 8 weeks following induction of hyperglycemia (Figure 3) showed that DM animals and/or TUDCA treatment had no effect on the overall appearance of ERG waveforms. Compared to other early treatment groups, DM+Veh animals had reduced a-wave amplitudes (Figure 4A, two-way repeated measures ANOVA, F_12,298_ = 4.121, main effect of group *p* < 0.001), increased a-wave implicit times (Figure 4C, two-way repeated measures ANOVA, F_3,170_ = 3.34, main effect of group *p* = 0.006), reduced b-wave amplitudes (Figure 4E, two-way repeated measures ANOVA, F_12,282_ = 2.421, main effect of group *p* < 0.001), and increased b-wave implicit times (Figure 4D, two-way repeated measures ANOVA, F_12,282_ = 2.46, main effect of group *p* < 0.001). OP2 amplitudes were similar between groups (Figure 5A). OP2 implicit times were significantly faster for the DM+TUDCA group compared to DM+Veh (Figure 5C; two-way repeated measures ANOVA F_12,212_ = 3.541, interaction effect *p* < 0.001). DM+TUDCA OP2 values were statistically indistinguishable from those of controls (Figure 5A,C).

In contrast, late treatment DM+TUDCA animals did not exhibit the same degree of retinal functional protection as early treatment DM+TUDCA. At week 8 post-STZ, both diabetic groups had significantly decreased a-wave (Figure 4B, two-way repeated measures ANOVA, F_12,243_ = 2.416, main effect *p* < 0.001) and b-wave amplitudes (Figure 4F, two-way repeated measures ANOVA, F_12,219_ = 2.416, main effect *p* < 0.001) when compared to their Ctrl counterparts. There were no significant differences in the amplitudes or implicit times of a-waves and b-waves between DM+Veh and DM+TUDCA groups (Figure 4B,D,F,H). OP2 amplitudes in the late treatment groups were not different (Figure 5A,B), while in the early treatment groups, OP2 implicit times were significantly slower with dim flash stimuli in the DM+Veh vs. DM+TUDCA groups (Figure 5C, two-way repeated measures ANOVA, F_12,282_ = 2.421, main effect of group *p* < 0.001). To further evaluate the effects of TUDCA, the change in OP2 implicit times between the 4 and 8 weeks post-STZ timepoints were plotted and slopes calculated. DM+TUDCA groups with either early and late treatment had negative slopes across time, indicating improved decreased implicit times with treatment (Figure 5E, early treatment slope, −0.38 ± 0.63; Figure 5F, late treatment slope, −0.54 ± 0.44), while DM+Veh groups showed increasing times (Figure 5E,F), although these trends did not reach statistical significance.

## 4. Discussion

In this study, we found that systemic treatment with TUDCA protected against retinal and visual dysfunctions in an STZ-induced Type 1 diabetic mouse model, which is consistent with observations in other disease models [9,10,16,17,29,41]. Furthermore, we demonstrated that the timing of treatment is a significant factor in TUDCA efficacy. TUDCA treatment starting one week post-induction of hyperglycemia provided a significant degree of neuroprotection, while delaying the treatment by mere 2 weeks reduced that effect.

Since treatment of early stage DR was a focus of this project, it was taken into consideration that the definition of early stage DR covers a broad timeframe in the overall course of the disease. Thus, we examined how the timing of treatment initiation affected overall neuroprotective outcomes. Visual function deficits, measured via optomotor response (OMR), were detectable in rodent models of DR as early as 3 weeks after inducing hyperglycemia [6,32], and retinal function deficits, measured via electroretinogram (ERG), were detectable as early as 4 weeks [6,32]. However, vascular abnormalities were not detectable until 6 months after initial hyperglycemia [42]. In order to model the use of TUDCA as an interventional treatment after a measurable loss of visual function, we initiated TUDCA treatment after 3 weeks of hyperglycemia (late treatment). Additionally, TUDCA has been investigated as a dietary supplement in several clinical trials for liver disease [43,44] and diabetes mellitus [45]. Thus, to model neuroprotective effects of TUDCA given as a supplemental therapy for diabetes mellitus before any detectable visual deficits, we treated a second group of diabetic animals with TUDCA after 1 week of hyperglycemia (early treatment).

Early TUDCA treatment in diabetic animals was associated with an increased performance on OMR thresholds and improved ERG parameters. Our results agree with previous studies investigating TUDCA and DR that show protective effects of TUDCA treatment beginning 1 day after hyperglycemia [29,46]. Late TUDCA treatment, on the other hand, seemed to demonstrate a much lower degree of protection across visual and retinal function assessments. However, a potential caveat in observing the decreased magnitude of neuroprotection with late treatment TUDCA is that the DM+Veh group in our late treatment experiment did not fully replicate the contrast sensitivity threshold declines and the ERG implicit time delays shown in both the early treatment cohort and previous studies [6,32]. This may be due to a variation across the cohorts as well as differences between individual animals. Nevertheless, the difference in outcomes between the early and late treatment cohorts did appear to indicate that treatment timing may play a role in determining the magnitude of TUDCA’s neuroprotective effects.

The mechanisms by which TUDCA acts are diverse and, therefore, it has been investigated as a potential treatment for a wide variety of disorders, including, but not limited to, neurodegenerative conditions. Bile acids in general have long been considered to be important regulators of digestion, particularly playing a role in fat metabolism [47]. Additionally, the bile acid specific receptor, farnesoid X receptor, is reported to be crucial for maintaining glucose homeostasis [48]. This is consistent with our observation that diabetic animals treated with TUDCA displayed significantly lower blood glucose levels when compared to their diabetic counterparts that received vehicle, especially in the early TUDCA treatment experiment.

In the retina, TUDCA has been shown to act on multiple cell types, through anti-apoptotic, anti-inflammatory, and antioxidant mechanisms [26]. For example, TUDCA has been reported to increase phagocytosis [49] and reduce oxidative stress in rod outer segments [50]. The rod outer segments are the sites of reactive oxygen species production and accumulation, which have been implicated as one of the pathogenic mechanisms in DR [51]. Thus, TUDCA may reduce oxidative stress in the rod photoreceptors and improve retinal function. The multiple molecular pathways through which TUDCA acts, likely contributes to its efficacy as a neuroprotective treatment, but also increases the complexity of the question as to how exactly it serves to rescue visual function.

Limitations of this study include a lack of outcome measures to provide insight into the molecular mechanisms of TUDCA neuroprotection. Testing animals at later timepoints and incorporating vascular assessments, such as optical coherence tomography angiography or VEGF levels, would have provided further evidence of TUDCA’s effect on vascular components of DR.

While we initially hypothesized that TUDCA intervention would be neuroprotective regardless of treatment onset, these results suggest that TUDCA may be more effective in its capacity as a neuroprotective treatment if drug intake begins as soon as diabetes is diagnosed, rather than administered as an acute treatment for diabetic retinopathy. Though TUDCA is being tested as a therapy for diabetes-associated conditions in four clinical trials [40,41,45,46], this finding may influence future clinical trials, highlighting the need to address questions pertaining to the timing of initiation of treatment, treatment interval, dosing, and assessment of impact on visual function. Additionally, this study assessed the functional outcomes of two different treatment timings while controlling for the overall time that animals were subjected to hyperglycemia. Perhaps future studies should control for the total duration of TUDCA treatment, regardless of treatment timing.

As noted, TUDCA is already being tested in clinical trials as a potential treatment for diabetes, as well as other neurodegenerative diseases such as amyotrophic lateral sclerosis (ALS). However, of the four ongoing or completed diabetes-focused clinical trials [40,41,45,46], outcome measures primarily included markers such as insulin levels, adipose tissue signaling, vascular endothelial factors, etc. Thus, there is a missed opportunity to explore functional outcomes, including vision. The literature demonstrates extensively that early retinal changes and neuronal damage precede vascular changes in diabetes [3,4,5,6,7,8,32], and since existing clinical trials do not focus on the visual outcomes of diabetes, there may be valuable data that were simply not collected. This study, therefore, emphasizes the importance of measuring visual function as an additional outcome parameter in future TUDCA clinical trials.

## Data Availability

The data presented in this study are available upon request from the corresponding author.
